# Comparison of the HER2, estrogen and progesterone receptor expression profile of primary tumor, metastases and circulating tumor cells in metastatic breast cancer patients

**DOI:** 10.1186/s12885-016-2587-4

**Published:** 2016-07-25

**Authors:** Bahriye Aktas, Sabine Kasimir-Bauer, Volkmar Müller, Wolfgang Janni, Tanja Fehm, Diethelm Wallwiener, Klaus Pantel, Mitra Tewes, on behalf of the DETECT Study Group

**Affiliations:** 1 Department of Gynecology and Obstetrics, University of Duisburg-Essen, Essen, Germany; 2 Department of Gynecology and Obstetrics, University Hospital Hamburg-Eppendorf, Hamburg, Germany; 3 Department of Gynecology and Obstetrics, University Hospital Ulm, Ulm, Germany; 4 Department of Gynecology and Obstetrics, University Hospital Duesseldorf, Duesseldorf, Germany; 5 Department of Gynecology and Obstetrics, University Hospital Tuebingen, Tuebingen, Germany; 6 Institut of Tumor Biology, Center of Experimental Medicine, University Hospital Hamburg-Eppendorf, Hamburg, Germany; 7 Department of Internal Medicine (Cancer Research), University Hospital Essen, Essen, Germany

**Keywords:** Metastatic breast cancer, Biopsy, Metastases, Receptor expression profile, CTC, Circulating tumor cells

## Abstract

**Background:**

The expression of HER2, estrogen (ER) and progesterone (PR) receptor can change during the course of the disease in breast cancer (BC). Therefore, reassessment of these markers at the time of disease progression might help to optimize treatment decisions. In this context, characterization of circulating tumor cells (CTCs) could be of relevance since metastatic tissue may be difficult to obtain for repeated analysis. Here we compared HER2/ER/PR expression profiles of primary tumors, metastases and CTCs.

**Methods:**

Ninety-six patients with metastatic BC from seven University BC Centers in Germany were enrolled in this study. Blood was obtained at the time of first diagnosis of metastatic disease or disease progression and analyzed for CTCs using the *AdnaTest BreastCancer* (QIAGEN Hannover GmbH, Germany) for the expression of EpCAM, MUC-1, HER2, ER and PR. HER2 expression on CTCs was additionally assessed by immunocytochemistry using the CellSearch® assay.

**Results:**

The detection rate for CTCs using the AdnaTest was 43 % (36/84 patients) with the expression rates of 50 % for HER2 (18/36 patients), 19 % for ER (7/36 patients) and 8 % for PR (3/36 patients), respectively. Primary tumors and CTCs displayed a concordant HER2, ER and PR status in 59 % (*p* = 0.262), 39 % (*p* = 0.51) and 44 % (*p* = 0.62) of cases, respectively. For metastases and CTCs, the concordance values were 67 % for HER2 (*p* = 0.04), 43 % for ER (*p* = 0.16) and 46 % for PR (*p* = 0.6). Using the CellSearch® assay, the CTC-positivity rate was 53 % (42/79 patients) with HER2 expressed in 29 % (12/42) of the patients. No significant concordance (58 % and 53 %) was found when HER2 on CTCs was compared with HER2 on primary tumors (*p* = 0.24) and metastases (*p* = 0.34). Interestingly, primary tumors and metastases were highly concordant for HER2 (84 %, *p* = 1.13E-08), ER (90 %, *p* = 3.26E-10) and PR (83 %, *p* = 2.09E-09) and ER-and PR-positive metastases were significantly found to be of visceral origin (*p* = 0.03, *p* = 0.02).

**Conclusion:**

Here we demonstrate that the molecular detection of HER2 overexpression in CTC is predictive of the HER2 status on metastases. Detailed analysis of ER and PR expression rates in tissue samples and CTCs may provide useful information for making treatment decisions.

## Background

In primary and metastatic breast cancer (MBC), tumors are usually analyzed for the presence or absence of the estrogen receptor (ER), the progesterone receptor (PR), and for amplification of HER-2, and the results of these analyses direct the types of treatment that patients receive. In MBC, patients may be treated with systemic therapy (chemotherapy, biological therapy, targeted therapy, hormonal therapy), local therapy (surgery, radiation therapy), or a combination of these treatments. The choice of treatment generally depends on the characteristics of the primary tumor because metastatic tissue is often difficult to obtain. Notably, HER-2 as well as ER/PR were shown to be differentially expressed between the primary tumor and corresponding metastases in up to 48 % which might lead to ineffective treatment in the absence of the respective marker [1–6]. Therefore, reassessment of these markers at the time of disease progression might help to optimize treatment decisions. Although biopsies from most metastatic sites may be obtained by the use of imaging and interventional radiology on a routine basis, these techniques are invasive and may pose some discomfort or may result in complications. Thus, a blood based biomarker would be desirable to bypass these problems.

In this regard, circulating tumor cells (CTCs) would be an ideal ‘surrogate tissue’ to identify prognostic and predictive factors that will help in selecting the optimal therapeutic strategy for each individual patient in case that metastatic tissue is not available.

Our study group has already demonstrated that ER and PR were differentially expressed between primary tumor and CTCs in MBC [7]. It was the purpose of the present study to compare the HER2/ER/PR expression profile of primary tumor and metastases, primary tumor and CTCs as well as metastases and CTCs. To our knowledge, it is the first study comparing histopathological and molecular findings between primary tumor, metastases and CTCs.

## Methods

### Patients and study design

A total of 96 MBC cancer patients, from seven University Breast Cancer Centers [Essen (*n* = 62), Düsseldorf (*n* = 6), Erlangen (*n* = 3), Hamburg (*n* = 10), Heidelberg (*n* = 5), Muenchen (*n* = 3), Regensburg (*n* = 2) and Tuebingen (*n* = 5)] in Germany were enrolled in this prospective open non-randomized study from 12/2007 until 04/2009. In general, most patients (69 %) had ductal breast cancer, moderately and poorly differentiated tumors were predominant. 73 % of the primary tumors were ER-, 55 % were PR-positive and 33 % had an overexpression of HER2 (Dako score 3+). Biopsies of metastases were taken from visceral (63 %) and non-visceral sites (37 %). Patients received different chemotherapeutic treatments in different lines of metastatic settings including anthracyclines, taxanes, capecitabine, vinorelbine and 5-FU or endocrine treatment including Tamoxifen, aromatase inhibitors and Fulvestrant (data not shown). CTCs from these patients were analyzed for ER/PR/HER2 expression during palliative therapy to compare these results with receptor expression on the primary tumor and metastases.

### Eligibility criteria

The eligibility criteria were as follows: Epithelial invasive carcinoma of the breast with distant metastatic disease (M1), age ≥ 18 years, first diagnosis of metastatic disease or disease progression (before start of new treatment regimen). Prior adjuvant treatment, radiation or any other treatment of metastatic disease were permitted.

Exclusion criterion was secondary primary malignancy (except in situ carcinoma of the cervix or adequately treated basal cell carcinoma of the skin). Blood was drawn before the start of a new line of therapy. A web-based databank was designed for data management and online-documentation (www.detetct-study.de). All specimens were obtained after written informed consent and collected using protocols approved by the institutional review board (2007/B01).

### Enrichment and molecular characterization of CTCs using the AdnaTest BreastCancer Kits

Two 5 ml EDTA blood samples were collected for isolation of CTCs using the AdnaCollect blood collection tubes (QIAGEN Hannover GmbH, Langenhagen, Germany) and stored at 4 °C until further examination. In-house samples were processed immediately or not later than 4 h after blood withdrawal, shipped samples were processed within 24 h. Establishment and validation of the AdnaTest BreastCancer assay has been described in detail elsewhere [7–9].

Briefly, all samples were subjected to immunomagnetic enrichment of CTCs using the AdnaTest *BreastCancerSelect* kit (QIAGEN Hannover GmbH, Langenhagen, Germany) followed by RNA isolation and subsequent gene expression analysis [EpCAM (GA733-2), MUC-1, HER2] by reverse transcription and Multiplex-PCR (polymerase chain reaction) in separated tumor cells using the The AdnaTest *BreastCancerDetect* (QIAGEN Hannover GmbH, Langenhagen, Germany) according to the instructions provided with the kit. Expression of ER and PR was assessed in an additional single-plex RT-PCR. Visualization of the PCR fragments was carried out with a 2100 Bioanalyzer using the DNA 1000 LabChips (Agilent Technologies) and the Expert Software Package (version B.02.03.SI307) both Böblingen, Germany. The primers generate fragments of the following sizes: GA 733–2: 395 base pairs (bp), MUC1: 293 bp, HER2: 270 bp, PR: 270 bp, ER: 305 bp, and actin: 114 bp.

#### Evaluation of data

The test is considered positive if a PCR fragment of at least one tumor associated transcript (MUC-1, GA 773–2 or HER2) is clearly detected. Peaks with a concentration of > 0.15 ng/μl are positive for the transcripts GA733-2, MUC1 and HER2. Peaks that are not detected at the above setting are negative (concentration of < 0.15 ng/μl). Peaks with a concentration of > 0.60 ng/μl are positive for the ER transcript and the PR expression is considered positive when the transcript is detected without applying any cut-off.

### Determination of HER2-expression using the CellSearch assay

Two 7.5 ml blood samples were collected into CellSave tubes (Veridex Inc.) for the CellSearch assay and sent at room temperature based on the manufacturer’s recommendation. Blood samples not processed within 96 h for the CellSearch assay were discarded. A validation study demonstrated that the samples could be stored and transported (up to 72 h) and showed high inter- and intra-assay concordance of the results in a multicenter setting [10].

In brief, CTCs are captured from peripheral blood by anti-EpCAM-antibody-bearing ferrofluid and identified by cytokeratin-positivity, negativity for the leukocyte common antigen CD45 and DAPI staining to ensure the integrity of the nucleus. HER2 expression of CTCs was characterized within the Cell Search system by addition of a FITC (Fluorescein isothiocyanate)-labeled anti-HER2 antibody (CellSearch^©^ tumor phenotyping reagent HER2/neu, Veridex, Raritan, NJ) as described previously [11]. The intensity of the HER2-specific immunofluorescence was categorized as negative (0), weak (1+), moderate (2+) and strong (3+). CTCs were considered HER2 positive if at least one CTC had a strong HER2 staining (3+).

### Immunohistochemical analysis of the primary tumor and metastases

The ER, PR and HER2 status of the primary tumor was obtained from the patients` charts. In all participating centers, the HER2 status has been determined by the HERCEP™ test (Dako, Glostrup, Denmark) and/or the Pathvysion-kit (HER2/neu) (Vysis, Downers Grove, IL).

All pathology laboratories had participated in ring experiments and were certified laboratories for ER, PR and HER2 detection. A central review of the ER, PR and HER2 status of the primary tumor as well as the metastases was, therefore, not performed.

### Statistical analysis

Concordance of the results between the different methods [AdnaTest (HER2;ER;PR), CellSearch (HER2) and tissue IHC for HER2, ER and PR] was evaluated using cross tabulation combined with Fisher’s exact-test. The comparison of the primary tissue and the metastatic tissue phenotype with regards to HER2, ER and PR was analyzed accordingly. WinSTAT® for Microsoft®Excel version 2012.1 (www.winstat.de) was used for the statistical calculations. Null hypothesis of discordant results was rejected when *p*-values were ≤ 0.05.

## Results

### Detection of CTCs

**Fig. 1:**
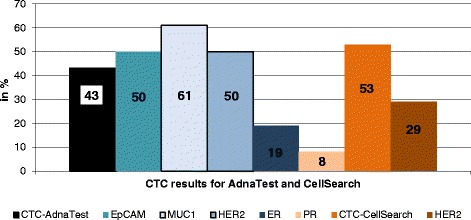
Results for CTCs obtained by the AdnaTest Breast Cancer and the CellSearch Assay

The detection rate for CTCs as determined by the AdnaTest and the CellSearch assay are demonstrated in Fig. 1. The AdnaTest could be applied in 84/96 patients (88 %) and resulted in an overall CTC detection rate of 43 % (36/84 patients) with the expression of 50 % (18/36 patients) for HER2 and EpCAM, 61 % for MUC-1 (22/36 patients), 19 % for ER (7/36 patients) and 8 % for PR (3/36 patients), respectively. Applying the CellSearch® assay for CTC detection in 79/96 (82 %) of patients, the CTC-positivity rate was 53 % (42/79 patients) with the expression rate of 29 % for HER2 (12/42 patients). Since the CellSearch system is based on immunomagnetic EpCAM capturing, a direct comparison of EpCAM-positive CTCs as detected by both test systems was performed. A comparison for EpCAM was feasible in 38 patients. The AdnaTest *BreastCancer* only detected 8 of 38 EpCAM-positive cases as evaluated by CellSearch. On the other hand, in the 37 CellSearch-negative cases, the AdnaTest detected 15 positive cases with the expression rates of 40 % for EpCAM and HER2 (both 6/15 patients) and 76 % for MUC-1 (11/15 patients), data not shown.

### Comparisons of expression profiles on CTCs with those on tissue samples

Comparisons of the expression profiles of ER, PR and HER2 on CTCs with those on tissue samples were only performed in CTC-positive patients. A comparison for HER2 was done applying the CellSearch® assay and the AdnaTest *Breast Cancer*. Due to technical requirements of both assays, a comparison of ER and PR was only feasible using the AdnaTest *BreastCancer*. All comparison studies are documented in Table 1.

**Table 1:** Comparisons of expression profiles on CTCs with those on tissue samples

	Primary Tumor	Metastases	*p*-value Concordance (C)	Primary Tumor	CTCs	*p*-value Concordance (C)	Metastases	CTCs	*p*-value Concordance (C)
Overall	96 (100 %)	96 (100 %)		36 (100 %)	36 (100 %)		36 (100 %)	36 (100 %)	
ER Status									
Negative Positive Unknown	25 (26 %) 70 (73 %) 1 (1 %)	24 (25 %) 64 (67 %) 8 (8 %)	*P* = 3,26E-10 C = 90 %	11 (31 %) 25 (69 %) 0 (0 %)	31 (86 %) 5 (14 %) 0 (0 %)	*P* = 0.51 C = 39 %	10 (28 %) 25 (69 %) 1 (3 %)	31 (86 %) 5 (14 %) 0 (0 %)	*P* = 0.16 C = 43 %
PR Status									
Negative Positive Unknown	42 (44 %) 53 (55 %) 1 (1 %)	44 (46 %) 44 (46 %) 8 (8 %)		15 (42 %) 21 (58 %) 0 (0 %)	33 (92 %) 3 (8 %) 0 (0 %)		15 (28 %) 20 (69 %) 1 (3 %)	33 (92 %) 3 (8 %) 0 (0 %)	
			*P* = 2,09E-09			*P* = 0.62 C = 44 %			*P* = 0.6 C = 46 %
			C = 83 %						
HER 2 Status									
Negative Positive Unknown	55 (57 %) 32 (33 %) 9 (10 %)	53 (55 %) 38 (40 %) 5 (5 %)		20 (55 %) 14 (39 %) 2 (6 %)	18 (50 %) 18 (50 %) 0 (0 %)	AdnaTest *P* = 0.26 C = 59 % Cellsearch	22 (61 %) 14 (39 %) 0 (0 %)	18 (50 %) 18 (50 %) 0 (0 %)	AdnaTest *P* = 0.0429 C = 67 % Cellsearch
			*P* = 1,13E-08 C = 84 %			*P* = 0,41 C = 58 %			*P* = 0,52 C = 53 %

Applying the AdnaTest *BreastCancer*, primary tumors and CTCs displayed a concordant HER2, ER and PR status in 59 % (*p* = 0.262), 39 % (*p* = 0.51) and 44 % (*p* = 0.62) of cases, respectively. For metastases and CTCs, the concordance values were 67 % for HER2 (*p* = 0.04), 43 % for ER (*p* = 0.16) and 46 % for PR (*p* = 0.6). Interestingly, in 26/36 patients with ER/PR-positive metastases, CTCs were positive in 27 % of cases and in the other 10 ER/PR-negative patients, the concordance was 100 % (*p* = 0.066).

Applying the CellSearch® assay, no significant concordance (58 % and 53 %) was found when HER2 status on CTCs was compared with HER2 expression on primary tumors (*p* = 0.41) and on metastases (*p* = 0.52).

Comparing the expression of the predictive markers on primary tumor and metastases, a high concordance was displayed for ER (90 %, *p* = 3.26E-10), PR (83 %, *p* = 2.09E-09) and HER2 (84 %, *p* = 1.13E-08). These results were confirmed when concordances for ER, PR and HER2 were only calculated in CTC-positive samples (ER: *p* = 7.2E-10; PR: *p* = 6.23E-10 and HER2: *p* = 0.001).

### Direction of concordance/discordance in the expression of ER, PR and HER2

As already described above, these analyses were only feasible using the results obtained with the AdnaTest *Breast Cancer*. As apparent from Table 2, a loss of receptor expression on CTCs can be seen for ER and PR when compared to the expression on primary tumors and on metastases. In contrast, although not significant, a trend for vice versa behaviour with regard to HER2 expression can be obtained for a substantial number of patients.

**Table 2:** Direction of the concordance/discordance in the expression of ER, PR and HER2

a) ER		Tumor ER-	Tumor ER+
	AdnaTest ER-	10	21
	AdnaTest ER+	1	4
		Fishers exact Test: *p* = 0,51	
		Metastases ER-	Metastases ER+
	AdnaTest ER-	10	20
	AdnaTest ER+	0	5
		Fishers exact Test: *p* = 0,16	
		Metastases ER-	Metastases ER+
	Tumor ER-	9	19
	Tumor ER+	1	2
		Fishers exact Test: *p* = 7,2E-6	
b) PR		Tumor PR-	Tumor PR+
	AdnaTest PR-	14	19
	AdnaTest PR+	1	2
		Fishers exact Test: *p* = 0,62	
		Metastases PR-	Metastases PR+
	AdnaTest PR-	14	18
	AdnaTest PR+	1	2
		Fishers exact Test: *p* = 0,60	
		Metastases PR-	Metastases PR+
	Tumor PR-	13	2
	Tumor PR+	2	18
		Fishers exact Test: *p* = 6,23E-6	
c)HER2		Tumor HER2-	Tumor HER2+
	AdnaTest HER2-	12	6
	AdnaTest HER2+	8	8
		Fishers exact Test: *p* = 0,043	
		Metastases HER2-	Metastases HER2+
	AdnaTest HER2-	14	4
	AdnaTest HER2+	8	10
		Fishers exact Test: *p* = 0,043	
		Metastases HER2-	Metastases HER2+
	Tumor HER2-	17	3
	Tumor HER2+	4	10

### Influence of the type of metastatic lesion on concordance

Table 3 illustrates the expression of predictive markers with regard to visceral (ML1) and bone (ML2) metastasis. Although not significant, visceral metastasis is more likely found in ER- as well as PR-positive tumors whereas no difference can be obtained for HER2. Interestingly, when these analyses were performed for metastases, ER- and PR-positive metastases significantly were found to be of visceral origin (*p* = 0.03; *p* = 0.02) whereas no trend was seen for HER2.

**Table 3:** Expression of predictive markers on primary tumor and metastases with regard to visceral (ML1) and bone (ML2) metastasis

	ML1	ML2
Tumor ER-	6	8
Tumor ER+	32	15
	Fischers exact Test: *p* = 0,08	
	ML1	ML2
Tumor PR-	13	13
Tumor PR+	25	10
	Fishers exact Test: *p* = 0,08	
	ML1	ML2
Tumor Her2-	22	15
Tumor Her2+	11	7
	Fishers exact Test: *p* = 0,9	
	ML1	ML2
Metastases ER-	6	9
Metastases ER+	33	14
	Fishers exact Test: *p* = 0,03	
	ML1	ML2
Metastases PR-	16	16
Metastases PR+	23	7
	Fishers exact Test: *p* = 0,02	
	ML1	ML2
Metastases Her2-	23	16
Metastases Her2+	16	7
	Fishers exact Test: *p* = 0,4	

## Discussion

In MBC, the choice of therapy generally depends on the size, location, and number of metastatic sites whereas the decision to administer antihormonal- and/or HER2-targeted therapy depends on the expression of these markers on the primary tumor since metastatic tissue is often difficult to obtain. However, several BC studies have indicated that the expression of HER2, ER and PR can change during course of disease [1–6, 12–30]. Therefore, reassessment of the predictive markers at the time of disease progression might help to optimize treatment decisions. In this context, characterization of CTCs could be of relevance in the future.

Here we demonstrate that the molecular detection of HER2 overexpression in CTCs using the AdnaTest *BreastCancer* is able to significantly predict the HER2 status on metastases. However, for ER/PR, a more detailed analysis of expression rates in tissue samples will be necessary to decide whether to use CTCs as a useful tool for treatment decisions. Interestingly, in contrast to some already published studies [1–6, 12–30], we could show that primary tumors and their metastases showed a highly significant concordance of the expression of predictive markers. Furthermore, ER- and PR-positive metastases significantly were found to be of visceral origin whereas no trend was seen for HER2 which has to be discussed in more detail.

In a prospective study, Simmons et al. demonstrated a discordant ER/PR and HER2 status in 40 % and 8 % of cases in more than half of the 40 patients analyzed who presented with new lesions suspicious for MBC. Consequently, therapeutic intervention was changed accordingly in 20 % of the patients who agreed to undergo biopsy not only to confirm their metastatic phenotype but also to have reassurance of receiving “targeted therapy” [3]. Another MBC study including 25 patients with liver metastases observed a discordant ER, PR and HER2 receptor status in 14.5 %, 48.6 % and 13.9 % of cases, respectively, which led to change in therapy in 12.1 % of patients [2]. With regard to HER2, subsequently published studies have reported discordant rates from 1 % to 24 % between primary tumor and metastases [1, 2, 12–29] and a study-level meta-analysis including 26 trials and about 2.500 patients, found a discordance rate for either HER2 loss or gain of 5.5 % [30, 31]. These findings are quite in opposite to our study, showing a significantly high concordance for these markers when comparing primary tumor and metastases.

A discordant expression of these receptors and the primary tumor and corresponding metastases and/or CTCs has already been demonstrated with a discordance between primary BC and HER2 expression on CTCs in the setting of disease recurrence at variable rates, with a gain of HER2 from 9 % to over 60 % in different studies [32–39]. The fact that ER and PR were differentially expressed between primary tumor and CTCs confirms the results of our previously published study demonstrating a loss of receptor expression on CTCs when compared with the expression on primary tumors [7]. These results can now be extended and confirmed for metastases and CTCs. However, the concordance for biomarker negativity seems to be higher in this study, although the number of these cases is quite small. In fact, the possibility of changes in receptor status during the course of tumor progression in triple-negative BC is very low, up to 8 %, despite changes in receptor positive BC with up to 40 % [40]. One could speculate that escape from antitumor therapy is more effective for CTCs when losing ER and PR on the surface.

In addition, the fact that ER- and PR-positive metastases significantly were found to be of visceral origin with a positive trend also documented in primary disease allows the hypothesis that CTC release as well as their downregulation of hormonal receptors might be recognized as a resistance mechanism to adjuvant endocrine therapy. As a consequence, CTCs released under therapy downregulate the therapeutic target during their phase of epithelial-mesenchymal transition (EMT) but recover ER/PR overexpression during the course of metastasis. This could fairly explain how hormone receptor positive visceral metastasis appear in significant concordance to the metastatic hormonal phenotype but also still seem to be positively correlated with the primary lesion. These results were not found for non-visceral metastases and we can only speculate that probably the different environment might influence the rate of receptor expression.

In our study, applying the AdnaTest *BreastCancer*, metastases and CTCs displayed a significantly concordant HER2 status in 67 % of cases whereas no significant concordance values could be shown for ER and PR. In contrast, applying the CellSearch® assay, no significant concordance was found when HER2 status on CTCs was compared to primary tumors and metastases. These findings might be explained by the different selection strategies of both assays. CellSearch® as an EpCAM-dependent assay might not detect CTCs that lost the EpCAM epitope and, therefore might result in false negatives with regards to HER2 overexpressing cells [41]. In contrast, the AdnaTest CTC enrichment method consists of an antibody mixture targeting EpCAM and MUC1, which might enable efficient CTC enrichment even in case EpCAM got lost.

However, CTCs are highly heterogeneous and using EpCAM-based capturing methods, it has been shown that this procedure is not able to detect the entire, highly heterogenous population of CTCs in MBC. In this regard, it has been demonstrated that these methods underestimate the most important subpopulations of CTCs involved in cancer dissemination, which often share EMT and stemness features [42–45]. In the current study, these subpopulations have not been analyzed which might explain discordant findings. Thus, despite the prognostic impact of CTC counts, molecular methods might complement these studies by improving the overall detection rate as well as sensitivity and, thus, permitting the assessment of genomic markers in CTCs of MBC patients as recently published [46].

From the clinical point of view, in a recent review of the literature, Turner and Di Leo concluded that the best management approach for receptor discordance between primary and metastatic disease is currently unknown, and the very limited evidence of alteration in clinical outcomes based on repeated biopsy does not seem to be strong enough to confirm that repeated biopsy is essential in every patient [47].

However, although these discrepancies have been demonstrated, the acquisition of tissue from metastases is not recommended as routine practice in any guideline. Thus, monitoring and phenotypic characterization of CTCs can provide new insights into the clonal selection of tumor cells under palliative therapies which may allow physicians to follow cancer changes over time and tailor treatment accordingly.

## Conclusion

To the best of our knowledge, this is the first study comparing HER2/ER/PR expression profiles of primary tumors, metastases and CTCs. Although we could show that primary tumors and their metastases showed a highly significant concordance of the expression of predictive markers, monitoring and a more comprehensive phenotypic characterization of CTCs will show whether CTCs can provide new insights into the clonal selection of resistant tumor cells under biological therapies. In this regard, the DETECT III phase III trial in Germany, comparing standard therapy alone versus standard therapy plus HER2 targeted therapy in patients with initially HER2-negative MBC and HER2-positive CTCs will probably answer that question. In this setting, patients with HER2-positive CTCs receive a targeted treatment option, noting that CTC detection and HER2 testing is performed by use of the CellSearch® assay (www.detetct-studien.de).
